# Neuroendocrine Adenoma of the Middle Ear: A Rare Histopathological Diagnosis

**DOI:** 10.1155/2016/9834750

**Published:** 2016-06-27

**Authors:** Zubair Hasan, Sam McGinness, Dakshika A. Gunaratne, Hedley Coleman, Winny Varikatt, Melville da Cruz

**Affiliations:** ^1^Department of Otolaryngology-Head & Neck Surgery, Westmead Hospital, Sydney, NSW 2153, Australia; ^2^Department of Tissue Pathology and Diagnostic Oncology, The Institute for Clinical Pathology and Medical Research (ICPMR), Sydney, NSW 2153, Australia

## Abstract

Neuroendocrine tumours occur throughout the body but are rare in the head and neck region and particularly rare in the middle ear. Clinical findings are often nonspecific and therefore pose a diagnostic challenge. Furthermore, the nomenclature of neuroendocrine tumours of the middle ear is historically controversial. Herein a case is presented of a middle ear adenoma in a 33-year-old patient who presented with otalgia, hearing loss, and facial nerve palsy. A brief discussion is included regarding the histopathological features of middle ear adenomas and seeks to clarify the correct nomenclature for these tumours.

## 1. Introduction

Neuroendocrine tumours of the middle ear, although rare, are a significant clinical entity and a diagnostic challenge. Middle ear neuroendocrine tumours were first reported in 1976 by Derlacki and Barney as an “adenomatous tumour” of the middle ear [1]. Since then the main point of controversy has been the nomenclature of such tumours and distinction of middle ear carcinoid tumours from adenomas or adenomatous tumours. Some authors consider them to be two distinct entities while others consider the tumours to be a spectrum of the same disease process. Our opinion is that they represent two distinct entities and we recommend the term neuroendocrine adenoma of the middle ear [2] in order to prevent perpetuation of this nosologic dilemma.

We describe the unique histopathological findings of a neuroendocrine adenoma of the middle ear in a 33-year-old male who presented to our institute with acute otalgia, hearing loss, and facial nerve palsy.

## 2. Case Presentation

A 33-year-old male presented to our emergency department with an acute onset of right sided facial nerve paralysis associated with a 4-day history of severe otalgia and intermittent tinnitus. He had a known background of a stable middle ear mass for which he was observed for 3 years at a different institution. He was otherwise fit and healthy with no comorbid illnesses or regular medications. Examination findings were consistent with an inflammatory middle ear effusion with a bulging tympanic membrane associated with right sided facial nerve palsy. Tuning fork tests demonstrated a conductive hearing loss in the affected ear. All other cranial nerves were intact. The patient was admitted under ENT for intravenous antibiotics and steroids. Within 72 hours of antibiotics administration, the otalgia subsided with improving facial nerve movement as well. He was discharged on oral antibiotics with follow-up in our outpatient department. With resolution of his middle ear infection, a pale red-pink mass was visible behind the tympanic membrane.

Further imaging demonstrated a middle ear mass with partial disruption of the facial canal. A subsequent angiogram demonstrated an avascular middle ear mass. Mixed hearing loss was demonstrated in the right ear on audiogram. The patient was electively taken to the operating theatre for a middle ear exploration and mastoidectomy with the intention of confirming the nature of the tumour and performing a subtotal removal. Intraoperative frozen section raised the possibility of a neuroendocrine tumour. The patient was successfully discharged on postoperative day 1. At 2 months follow-up postoperative hearing was preserved with normal facial nerve function and no evidence of recurrence.

Examination of histological sections stained with haematoxylin and eosin revealed small fragments of mucosa with underlying pieces of vital bone. The mucosa was surfaced by nonkeratinising stratified squamous epithelium which showed no epithelial dysplasia with no evidence of surface origin of the tumour. The underlying lamina propria was extensively infiltrated by an unencapsulated tumour. The tumour cells were arranged in small irregular nests and trabeculae with surrounding fibrosis. Occasional glandular structures were also identified. The individual tumour cells had scanty granular eosinophilic cytoplasm with hyperchromatic nuclei. Mitoses were not observed. There was no evidence of necrosis within the tumour. The tumour cells demonstrated positive staining for cytokeratin (AE1/AE3) with keratin 7 (CK7) staining the luminal cells in the glandular structures. The neuroendocrine immunohistochemical markers (CD56, chromogranin, and synaptophysin) were positive. No sustentacular cells were identified (S-100 negative) and the tumour had a low proliferation index of 3-4% (Ki-67).

## 3. Discussion

The so-called “carcinoid” tumour of the middle ear has previously been described by many names including neuroendocrine adenoma of the middle ear, adenomatous tumour of the middle ear, and adenocarcinoid tumours. We have opted for the term neuroendocrine adenoma of the middle ear as proposed by Thompson [2] based upon the morphologic appearance, known behaviour, and outcome for this rare tumour. Middle ear neuroendocrine tumours were first reported in 1976 by Derlacki and Barney as an “adenomatous tumour” of the middle ear [1]. Since then the main point of histopathologic controversy has been the nomenclature of such tumours and distinction between middle ear carcinoid tumours and adenomas or adenomatous tumours with some authors considering them two distinct entities while others consider the tumours within the same spectrum of disease.

Derlacki and Barney initially described the histological appearance of the tumour in 3 patients in their twenties as nests of cells with glandular epithelial appearance. The first case of “carcinoid tumour” in the middle ear which appears in the literature is described by Murphy et al. in 1980 who described neuroendocrine differentiation resembling an adenomatous tumour [3]. Neuroendocrine tumours typically occur in the lung or gastrointestinal tract where cells of neuroendocrine differentiation are present. However, in the middle ear where histological examination has not demonstrated native neuroendocrine cells, they are thought to arise from pluripotent stem cells [4].

With regard to nomenclature, we favour using the term neuroendocrine adenoma of the middle ear (NAME) as proposed by Thompson and Mills for this benign tumour over the term carcinoid tumour which by definition represents a well-differentiated neuroendocrine carcinoma with metastatic potential [2, 5]. The terminology of neuroendocrine tumours in the head and neck is controversial and confusing at best with the World Health Organisation suggesting use of the terms carcinoid, atypical carcinoid, and small cell carcinoma similar to that terminology used in lung pathology. Mills [5] on the other hand however favours the terms well-, moderately, and poorly differentiated neuroendocrine carcinomas to describe the different entities and the potential biologic behaviour of these uncommon lesions. Based upon long-term follow-up, these neoplasms of the middle ear are benign and have an indolent course supporting the fact that these adenomas of the middle ear exhibit neuroendocrine differentiation and that they should not be considered a carcinoid tumour.

**Figure 1:**
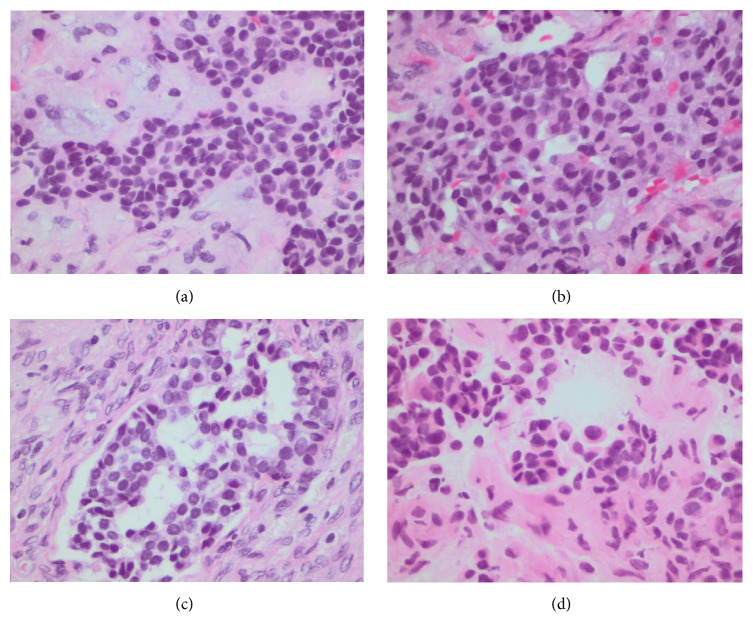
The tumour exhibits variable growth patterns including trabeculae (a), solid sheets (b), and glandular structures (c). The cells have eosinophilic cytoplasm with oval nuclei which may appear plasmacytoid in nature (d) (H&E).

**Figure 2:**
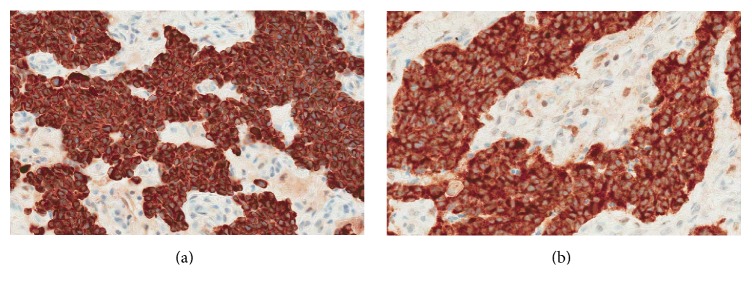
The tumour cells show immunoreactivity to a range of keratins including AE1/AE3 (a) and neuroendocrine markers such as synaptophysin (b) (Ventana systems).

Histologically NAME presents an unencapsulated tumour with similar cytomorphologic and staining patterns but different architectural growth patterns. They usually exhibit a dual cell population with growth patterns ranging from anastomosing cords, trabeculae to solid sheets, and glandular structures (Figure 1). The cells usually have eosinophilic cytoplasm that may be finely granular with oval nuclei that demonstrate little pleomorphism with “salt and pepper” chromatin, tiny inconspicuous nucleoli, and very scanty mitoses [2]. The glandular structures may contain amorphous secretion [6]. The tumour cells show immunoreactivity to a range of keratins including AE1/AE3 (Figure 2(a)), keratin 7, and neuroendocrine markers such as chromogranin and synaptophysin (Figure 2(b)). Ultrastructurally two cell types have been confirmed with apical cells that stain preferentially with keratin 7 and basal cells that contain neuroendocrine granules and which stain positively for the neuroendocrine markers.

Neuroendocrine adenomas of the middle ear occur most commonly in the 20–30-year age group. Nevertheless, adolescent cases have been described as well [7–9]. The most common presentation is unilateral hearing loss, which has been described as sensorineural [3, 10] or more commonly conductive [8, 11–15]. Mixed hearing loss [4] or even normal hearing [16] has also been demonstrated. Associated symptoms include tinnitus [15], transient facial nerve paralysis [17], aural fullness [8, 9, 15], otalgia [9], or otorrhoea [4, 15]. Symptoms of true carcinoid syndrome such as flushing and diarrhoea cause by released neurohormonal peptides are rare [12, 18].

Diagnosis is difficult and often not complete until histopathological examination has been undertaken. This is demonstrated in the literature with a number of cases initially managed surgically with a suspected diagnosis of cholesteatoma [19] or paraganglioma. This was the initial suspicion in our case as well. Imaging should be obtained commencing with CT scan of the involved region, although findings are usually nonspecific and generally demonstrate an isodense soft-tissue opacity in the middle ear occasionally with evidence of bony erosion.

Although the tumours are usually unencapsulated and may on occasion exhibit an “infiltrative growth pattern” and very rarely metastasise, they are considered benign since the other features of malignancy which would include cytologic pleomorphism, mitotic activity, and necrosis as well as perineural and lymphovascular invasion are not observed.

Patients should be followed up closely due to the possibility of recurrence and metastatic disease. Serial clinical examination should suffice with imaging indicated with suspicion of recurrence. Knerer et al. reported one case of recurrence for which a subtotal petrosectomy was indicated [20]. More recently Aoki et al. have also described a similar case with recurrence 7 years after radical tympanomastoidectomy which also required a petrosectomy [21]. Although considered a benign neoplasm, regional metastases have occurred in the cervical lymph nodes and parotid gland [22–25] and these regions should be assessed and neck dissection performed where required. Distant metastasis has also been described by Fundakowski et al. [26] in the iliac crest and cervical lymph nodes. Gaafar et al. had also previously described visceral hepatic metastasis. In this particular case, locally aggressive disease ultimately led to mortality with middle cranial fossa extension [19]. Metastasis occurred between 8 months and 43 years of follow-up and therefore long-term follow-up is necessary.

The majority of cases of middle ear neuroendocrine tumours reported in the literature were treated with radical mastoidectomy including resection of the ossicular chain where required. Further surgical treatment is indicated in the event of metastasis and has been reported in the literature with selective neck dissection. Parotid metastasis has been successfully managed with superficial parotidectomy. Adjuvant radiotherapy has been used in some cases in the literature; however, due to the paucity of available data, outcomes are difficult to measure and the impact of any radiotherapy administered is difficult to assess. In conclusion, although technically difficult, surgical resection of the tumour as well as the ossicles is the treatment of choice for NAME with excellent overall prognosis, a recurrence rate of only about 15% and only few documented cases of metastatic disease.
